# Memory-Based Multiagent Coevolution Modeling for Robust Moving Object Tracking

**DOI:** 10.1155/2013/793013

**Published:** 2013-06-16

**Authors:** Yanjiang Wang, Yujuan Qi, Yongping Li

**Affiliations:** College of Information and Control Engineering, China University of Petroleum, No. 66, Changjiang West Road, Economic and Technological Development Zone, Qingdao 266580, China

## Abstract

The three-stage human brain memory model is incorporated into a multiagent coevolutionary process for finding the best match of the appearance of an object, and a memory-based multiagent coevolution algorithm for robust tracking the moving objects is presented in this paper. Each agent can remember, retrieve, or forget the appearance of the object through its own memory system by its own experience. A number of such memory-based agents are randomly distributed nearby the located object region and then mapped onto a 2D lattice-like environment for predicting the new location of the object by their coevolutionary behaviors, such as competition, recombination, and migration. Experimental results show that the proposed method can deal with large appearance changes and heavy occlusions when tracking a moving object. It can locate the correct object after the appearance changed or the occlusion recovered and outperforms the traditional particle filter-based tracking methods.

## 1. Introduction

The problem of object tracking is often posed as that of estimating the trajectory of objects in an image plane as objects move in a scene [1]. Although considerable efforts have been made in establishing a robust tracking framework in the research literature, the problem still remains challenging when appearance abrupt changes or occlusions occur. To address these challenges, in the literature tremendous attempts have been made in characterizing appearance models which are able to handle appearance changes. In this context, most of the extant methods tend to apply a total model updating mechanism for template updating in which the initial template model is updated gradually based on the estimated information, for example particle filters (PF). However, if an object is heavily occluded or its appearance changes abruptly, the total model updating based PF (TMU-PF) will gradually deviate from the target.

Recently, a lot of modifications have been made for improving the performance of particle filters. For example, Zhou et al. [2] presented an approach that incorporated appearance-adaptive models to stabilize the tracker. They made three extensions: (a) an observation model arising from an adaptive appearance model, (b) an adaptive velocity motion model with adaptive noise variance, and (c) an adaptive number of particles. Li et al. [3] proposed a robust observation model to address appearance changes. Wang et al. [4, 5] developed an SMOG appearance model and an SMOG-based similarity measure to deal with appearance variations. Zhang et al. [6] embedded an adaptive appearance model into a particle filter to address the appearance changes and proposed an occlusion handling scheme to deal with occlusion situations. On the other hand, some researchers have incorporated other optimization algorithms into particle filer to enhance the performance. For example, in [7], CamShift was used into the probabilistic framework of particle filter as an optimization scheme for proposal distribution such that both the tracking robustness and computational efficiency are improved. Shan et al. [8] incorporated the mean-shift (MS) optimization algorithm into a particle filter framework to improve the sampling efficiency. Zhou et al. [9] presented a scale invariant feature transform (SIFT) based mean shift algorithm for object tracking, which improved the tracking performance of the classical mean shift and SIFT tracking algorithms in complicated real scenarios. Zhao and Li [10] applied particle swarm optimization (PSO) to find high likelihood areas where the particles could be distributed even though the dynamic model of the object could not be obtained. Zhou et al. [11] combined multiband Generalized Cross Correlation, KF, and Weighted Probabilistic Data Association within the particle filtering framework, which improves the performance of the algorithm in noisy scenarios. Most of the above methods applied a total model updating mechanism for template updating in which the initial template model is updated gradually based on the estimated information by particle filters. However, if an object is heavily occluded or its appearance changes abruptly, the total model updating based PF (TMU-PF) will gradually deviate from the target.

To tackle the drawback of the TMU-PF, Montemayor et al. [12] introduced memory strategies into PF to store the states of particles, which can deal with some occlusion situations. Mikami et al. [13] proposed a memory-based particle filter (MPF) to handle facial pose variation by predicting the prior distribution of the target state in future time steps. However, both of the methods are neither biologically motivated nor cognitively inspired. They just apply memory to store the states of particles and could not cope with situations with sudden changes.

It is well known that humans can track and recognize an object with little difficulty in the case of appearance changes and partial occlusions. This capability of human beings benefits from the human's memory system. When humans perceive something, the related information which is stored in their memory can be recalled. As a function of information retention organs in the brain, the mechanism of memory system has been extensively studied in neural science, biopsychology, cognitive science, and cognitive informatics [14, 15].

Inspired by the way humans perceive the environment, in this paper, we present a memory-based multiagent coevolution model for tracking the moving objects. The three-stage human brain memory mechanism is incorporated into a multiagent coevolutionary process for finding a best match of the appearance of the object. Each agent can remember, retrieve, or forget the appearance of the object through its memory system by its own experience. A number of such memory-based agents are randomly distributed nearby the located object region and then mapped onto a 2D lattice-like environment for predicting the new location of the object by their coevolutionary behaviors, such as competition, recombination, and migration. Experimental results show that the proposed method can deal with large appearance changes and heavy occlusions when tracking a moving object. It can locate the correct object after the appearance changed or the occlusion recovered.

The remainder of this paper is organized as follows. In Section 2, we will first propose the memory-based multiagent coevolution model including the definitions of each behavior involved.  Section 3 gives the detailed description of the memory modeling of an agent and the object appearance template updating process for each agent. Then the color object modeling and the proposed tracking algorithm are described in Section 4. Finally, the performance of our tracking algorithm is verified on different standard video sequences and some conclusions are summarized in Sections 5 and 6.

## 2. Memory-Based Multiagent Coevolution Modeling 

### 2.1. Memory-Based Multiagent Model

According to [16], an agent can be defined as an intelligent entity that resides in an environment and can act autonomously and collaboratively. It is driven by certain purposes and has some reactive behaviors. Based on this idea, many agent-based applications are reported during past years, such as image feature extraction [17], image segmentation [18], and optimization problems [19–24]. In our previous work [25, 26], we also proposed an evolutionary agent model for color-based face detection and location. 

In this paper, we will present a memory-based multiagent model (MMAM) for moving object tracking. Each agent represents a candidate target region in a video frame; it lives in a lattice-like environment, and its main task is to compete or cooperate with its neighbor agents to continuously improve its own fitness by exhibiting its behaviors.

**Figure 1:**
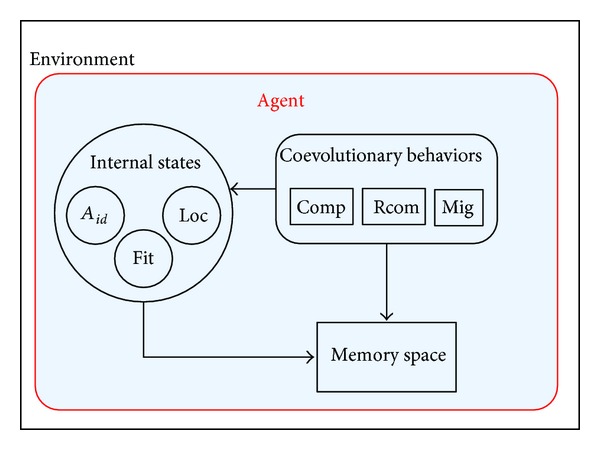
Memory-based agent model.

The schematic diagram of the proposed MMMA is shown in Figure 1.

More specifically, the memory-based multiagent model (MMAM) for object tracking can be defined as a 7-tuples: 〈*A*_*id*_, Loc, Fit, MS, Comp, Rcom, Mig〉. Where *A*_*id*_ denotes the identity of an agent; Loc represents the position of an agent in the image, that is, the center of a candidate target; Fit symbolizes its fitness, which is defined by the similarity between the candidate target and the object template; and MS = {USTMS, STMS, LTMS} is a set of hominine memory spaces of an agent for information storage, where USTMS, STMS, and LTMS stand for the ultrashort-term memory space, short-term memory space, and long-term memory space, respectively.

The above 4 parameters describe the internal states of an agent. While Comp, Rcom, and Mig describe the external coevolutionary behaviors of an agent, where Comp represents the competition behavior, Rcom denotes the recombination behavior, while Mig refers to the migration behavior.

**Figure 2:**
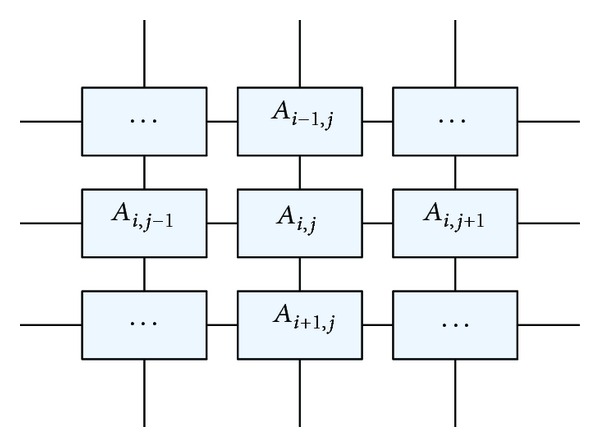
Model of the agent lattice.

Suppose all the agents inhabit in a lattice-like environment, *A*, which is called an agent lattice, as shown in Figure 2. Each agent is fixed on a lattice point and it can only interact with its 4 neighbors. The size of  *A*  is *N* × *N* and the agent located at (*i*, *j*) is denoted by *A*_*i*,*j*_, *i*, *j* = 1,2,…, *N*. Each agent can compete or cooperate with its 4 neighbors in order to improve its fitness.

The mapping process is described as follows.

First, it randomly generates *N* × *N* agents near the located object region at begining. The first generated agent is placed at *A*_1,1_, the second agent is placed at *A*_1,2_,…, the *N*th agent is placed at *A*_1,*N*_, the (*N* + 1)th agent is placed at *A*_2,1_,…, and the final agent (*N* × *N*)th is placed at *A*_*N*,*N*_. The neighbors of agent *A*_*i*,*j*_ are defined as *Nb*_*i*,*j*_ = {*A*_*i*−1,*j*_, *A*_*i*+1,*j*_, *A*_*i*,*j*−1_, *A*_*i*,*j*+1_}. For the agents at the four edges of the lattice, we define

(1) A0,j=AN,j,  Ai,0=Ai,N,AN+1,j=A1,j,  Ai,N+1=Ai,1.

According to the above definition, the neighbors of an agent on the lattice are not its real neighbors in the video image. Because each agent is generated randomly and can only evolve with its neighbors on the lattice-like environment, the mapping process can also be thought as a natural selection before their coevolution.

### 2.2. Multiagent Coevolutionary Behaviors

There are three coevolutionary behaviors for each agent, that is, competition, recombination, and migration. The three behaviors are defined as follows.

**Definition 1 (Comp (competition behavior))**

Comp means that an agent will contend with other agents for its survival.

For each agent *A*_*i*,*j*_, if Fit(*A*_*i*,*j*_) < Fit(*Nb* max_*i*,*j*_), where *Nb* max_*i*,*j*_ is the agent with maximum fitness among its 4 neighbors, then *A*_*i*,*j*_ will be replaced by the following:

(2) $$\begin{array}{c} A_{i,j}^{l}=\{\begin{array}{cc} \underline{l},\;\;\;\;\;\;\;\;\;\;\;\;\;(Nb\;\text{ma}{\text{x}}_{i,j}^{l}+U\left(-1,1\right)\;\; & \\ \;\;\;\;\;\;\;\;\;\;\;\;\;\;\;\;\;\times \left(Nb\;\text{ma}{\text{x}}_{i,j}^{l}-A_{i,j}^{l}\right))<\underline{l}, & \\ \bar{l},\;\;\;\;\;\;\;\;\;\;\;\;\;(Nb\;\text{ma}{\text{x}}_{i,j}^{l}+U\left(-1,1\right)\;\; & \\ \;\;\;\;\;\;\;\;\;\;\;\;\;\;\;\;\;\times \left(Nb\;\text{ma}{\text{x}}_{i,j}^{l}-A_{i,j}^{l}\right))>\bar{l}, & \\ Nb\;\text{ma}{\text{x}}_{i,j}^{l}+U\left(-1,1\right)\;\;\;\;\;\;\;\;\;\;\;\; & \\ \;\times \left(Nb\;\text{ma}{\text{x}}_{i,j}^{l}-A_{i,j}^{l}\right),\;\;\text{otherwise}, & \end{array} \end{array}$$

where *U*(−1,1) is a uniform random number in [−1,1], *l* denotes the location of agent *A*_*i*,*j*_ in the video frame, *l* = (*x*, *y*), $L=[\underline{l},\bar{l}]$ represents the whole searching space, that is, the video size, $\underline{l}=[\underline{x},\underline{y}]$, $\bar{l}=[\bar{x},\bar{y}]$.

**Definition 2 (Rcom (recombination behavior))**

Rcom means that an agent may exchange the *x* or *y* coordinate with other agents. It is similar to the crossover operator in genetic algorithms.

For each agent *A*_*i*,*j*_, given a recombination probability *P*_*r*_, if *U*(0,1) < *P*_*r*_, exchange the *x* or *y* coordinate of *A*_*i*,*j*_ and *Nb* max_*i*,*j*_, a new agent will be created, *Ar*_*i*,*j*_. If Fit(*A*_*i*,*j*_) > Fit(*Ar*_*i*,*j*_), *A*_*i*,*j*_ will continue to exist in the lattice; otherwise it will be replaced by the following:

(3) $$\begin{array}{c} Ar_{i,j}=\{\begin{array}{cc} \left(A_{i,j}^{x},Nb\;\text{ma}{\text{x}}_{i,j}^{y}\right), & U\left(0,1\right)<0.5,\\ \left(Nb\;\text{ma}{\text{x}}_{i,j}^{x},A_{i,j}^{y}\right), & \text{else}. \end{array} \end{array}$$

**Definition 3 (Mig (migration behavior))**

Mig means that an agent can move to another location by some random steps in the image other than the lattice it locates at. It is similar to the mutation operator in genetic algorithms.

For each agent *A*_*i*,*j*_, the migration behavior will occur according to a migration probability *P*_*m*_. if *U*(0,1) < *P*_*m*_, *A*_*i*,*j*_ will be replaced by the following:

(4) $$\begin{array}{c} Am_{i,j}^{l}=\{\begin{array}{cc} \underline{l}, & A_{i,j}^{l}+U^{l}\left(-10,10\right)<\underline{l},\\ \bar{l}, & A_{i,j}^{l}+U^{l}\left(-10,10\right)>\bar{l},\\ A_{i,j}^{l}+U^{l}\left(-10,10\right), & \text{otherwise}, \end{array} \end{array}$$

where *U*(−10,10) is a uniform random number in [−10,10]; that is, the migration steps are randomly generated within (−10, 10) pixels for *i* and *j*, respectively.

## 3. Memory Modeling for an Agent

### 3.1. Three-Stage Human Brain Memory Modeling for Appearance Updating

**Figure 3:**
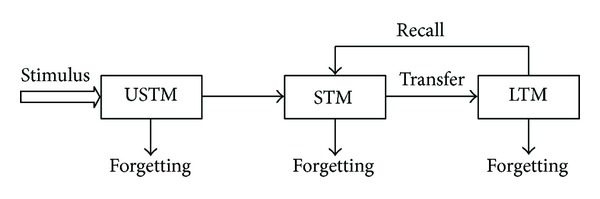
Three-stage human brain memory model.

As a faculty of information retention organs in the brain, memory has been intensively studied in psychology, neural science, and cognitive science, and several memory models have been proposed since the late 19th century. In 1890, James first divided the human memory into three components: after-image memory, the primary memory, and the secondary memory [27]. Atkinson and Shiffrin modeled the human memory as a sequence of three stages: the sensory memory, short-term memory, and long-term memory [28] (also known as the multistore model). Baddeley and Hitch proposed a multicomponent model of working memory where a central executive responsible for control processes and two slave systems providing modality-specific buffer storage [29]. Recently, Wang proposed a logical architecture of memories in the brain which includes four parts: (a) the sensory buffer memory; (b) the short-term memory; (c) the long-term memory; and (d) the action buffer memory [15, 30]. According to contemporary cognitive psychology, the popular model of a basic human brain memory includes three stages: ultrashort-term memory (USTM), short-term memory (STM), and long-term memory (LTM), as shown in Figure 3 [31].

Each stage includes three processes: (a) encoding, (b) storage, and (c) retrieval. “Encoding” (also referred to as registration) is the process of forwarding physical sensory input into one's memory. It is considered as the first step in memory information processing. “Storage” is the process of retaining information whether in the sensory memory, the short-term memory, or the more permanent long-term memory. “Retrieval” (also referred to as “recall”) is to call back the stored information in response to some cues for use in a process or activity.

The memorization process can be described as follows.

1. USTM is used to store the basic cognitive information.
2. STM, which in the recent literature has been referred to as working memory, is used to make decision. The information stored in STM includes the new information from USTM, the information processed in STM, or the information recalled from LTM. Therefore, STM can be considered as a complicated system for information storing and processing.
3. LTM is a library used to store experienced knowledge which can inspire the individual to recall every thing that had happened, cognize all kinds of models, and solve problems (e.g., tracking problems in our work).
4. Forgetting is a special function of memory which helps the information either not always recalled or not commonly used to be lost from memory.

**Figure 4:**
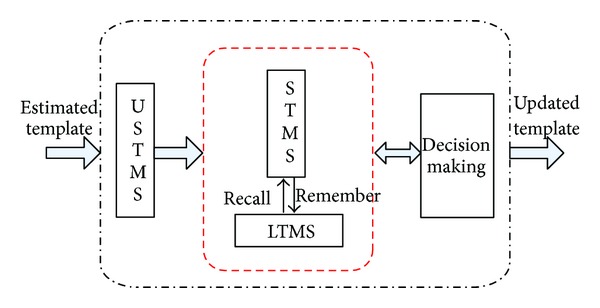
Three-stage memory model for appearance template updating.

According to the above three-stage human memory model, the appearance template updating model of an agent can be described as shown in Figure 4,

where the input of the model is the candidate template estimated by the Loc of an agent in the current video frame while the output is the updated template for prediction in the next frame. USTMS, STMS, and LTMS represent the three-stage memories, respectively. They are defined as follows.

**Definition 4 (memory space (MS))**

A 3-tuple which is used to store the current estimated appearance template and the past templates. Each element in MS is a memory space:

(5) $$\begin{array}{c} \text{MS}=\left\{\text{USTMS},\text{STMS},\text{LTMS}\right\}. \end{array}$$

**Definition 5 (USTMS)**

A one-element set for storing the estimated model *p* in the current video frame, which simulates the stage of ultrashort-term memory of human brain:

(6) $$\begin{array}{c} \text{USTMS}=\left\{p\right\}. \end{array}$$

**Definition 6 (STMS)**

A set of *K*_*s*_ temporary templates, which imitates the stage of short-term memory of human brain. Let *q*_*i*_ denote the *i*th template in STMS; then

(7) $$\begin{array}{c} \text{STMS}=\left\{q_{i},\;i=1,2,\ldots ,K_{s}\right\}. \end{array}$$

**Definition 7 (LTMS)**

A set of *K*_*l*_ remembered templates, which simulates the dynamic stage of the long-term memory of human brain. Let *q*_*Mj*_ stand for the *j*th remembered template in LTMS:

(8) $$\begin{array}{c} \text{LTMS}=\left\{q_{Mj},\;j=1,2,\ldots ,K_{l}\right\}. \end{array}$$

The templates stored in STMS include the estimated template transferred from USTMS, the updated templates in STMS, or the templates recalled from LTMS.

According to the theory of cognitive psychology, only the information which is stimulated repeatedly can be stored into LTMS. Therefore, we define a parameter *β* for each template in STMS to determine whether the templates in STMS can be stored into LTMS or not, where *β* is a counter indicating the number of successful matches. The bigger *β* is, the more probably the template can be stored into LTMS.

More specifically, for all *q*_*i*_ ∈ STMS, *i* = 1,2,…, *K*_*s*_, If *q*_*i*_.*β* > *T*_*M*_ (a predefined threshold), the template will be remembered and stored into LTMS.

The process of template updating can be briefly described as follows.

First, the estimated template of the current frame is stored into USTMS and checked against the current template in STMS (the first one). If they are matched, update the template; otherwise check against the remaining templates in STMS and then LTMS in turn for a match. If a match exists, it will be selected for the new template. Meanwhile the STMS and LTMS are updated by some behaviors, such as remembering, recall, and forgetting. These behaviors are defined as follows.

**Definition 8 (remembering)**

An action that a template is stored into LTMS.

If there is no match in STMS and LTMS, and the STMS is full and the last template in STMS (denoted by *q*_*K*_*s*__) is satisfied with *q*_*K*_*s*__.*β* > *T*_*M*_, then *q*_*K*_*s*__ will be remembered into LTMS and replaced by *q*_*K*_*s*_−1_. In such a circumstance, the estimated template will be reserved for the next estimation.

**Definition 9 (recall)**

An action that a matched template is loaded from LTMS.

If a match is found in LTMS, the matched template will be extracted and used as the current object template.

**Definition 10 (forgetting)**

An action that a template is removed from either of STMS or LTMS.

If the LTMS is full and *q*_*K*_*s*__.*β* > *T*_*M*_, the oldest template in LTMS will be forgotten in order to remember *q*_*K*_*s*__.

### 3.2. Detailed Description of Memory-Based Appearance Updating

According to the above model, the memory-based appearance template updating algorithm can be described as follows.

**Step 1 (Initialization)**

**Figure 5:**
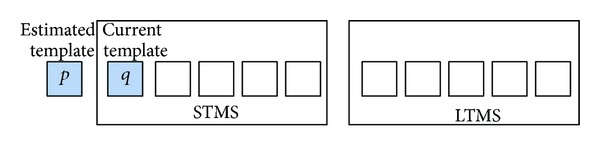
Initialization step.

For each agent, store the estimated template (candidate object) *p* into the USTMS and the current template *q* into the STMS; set *q*.*β* = 1 and the LTMS to be empty, where *p* and *q* are determined by the initial target region, as shown in Figure 5. It is worth mentioning that the STMS and LTMS will be filled up gradually after several time steps during tracking.

**Step 2**

Calculate the similarity coefficient *ρ* = *ρ*[*p*, *q*], if *ρ* > *T*_*dc*_, update the current object template by the following:

(9) $$\begin{array}{c} q=\left(1-\alpha \right)q+\alpha \cdot p,\\ q.\beta =q.\beta +1, \end{array}$$

where *T*_*dc*_ is a predefined threshold for current template matching and *α* is the updating rate.

**Step 3**

If *ρ* ≤ *T*_*dc*_, check against the remaining templates in STMS for a match, if

(10) $$\begin{array}{c} \rho \left[p,q_{i}\right]>T_{ds},\;i=1,\ldots ,K_{s}-1, \end{array}$$

update the matched template by the following:

(11) $$\begin{array}{c} q_{i}=\left(1-\alpha \right)\cdot q_{i}+\alpha \cdot p,\\ q_{i}.\beta =q_{i}.\beta +1, \end{array}$$

where *T*_*ds*_ is the threshold for template-matching in STMS.

**Figure 6:**
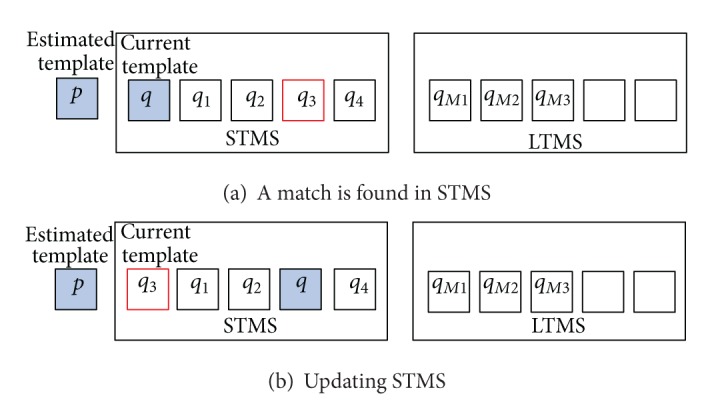
Illustration of updating process in STMS.

Then, exchange the current template and the matched one, as shown in Figure 6.

For example, if *q*_3_ is a matched template found in STMS (as shown in Figure 6(a)), then it will be moved to the top location in STMS and used as the current template, while the previous current template *q* will be moved to the original location of *q*_3_ as shown in Figure 6(b).

**Step 4**

**Figure 7:**
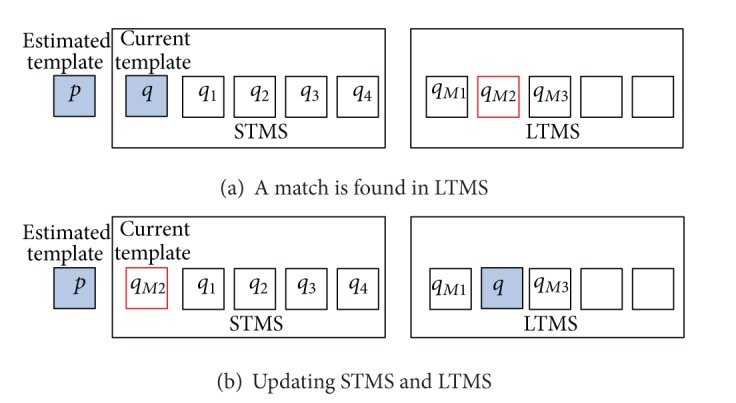
Illustration of recalling and remembering.

If *ρ*[*p*, *q*_*i*_] ≤ *T*_*ds*_, check in LTMS for a match, if

(12) $$\begin{array}{c} \rho \left[p,q_{Mj}\right]>T_{dl},\;j=1,\ldots ,K_{l}, \end{array}$$

where *T*_*dl*_ is the threshold for template-matching in LTMS. Then update the matched template by the following:

(13) $$\begin{array}{c} q_{Mj}=\left(1-\alpha \right)q_{Mj}+\alpha \cdot p,\\ q_{Mj}.\beta =q_{Mj}.\beta +1, \end{array}$$

and then recall the matched one to use as the new object template and remember the current template *q*, as shown in Figure 7.

**Step 5**

**Figure 8:**
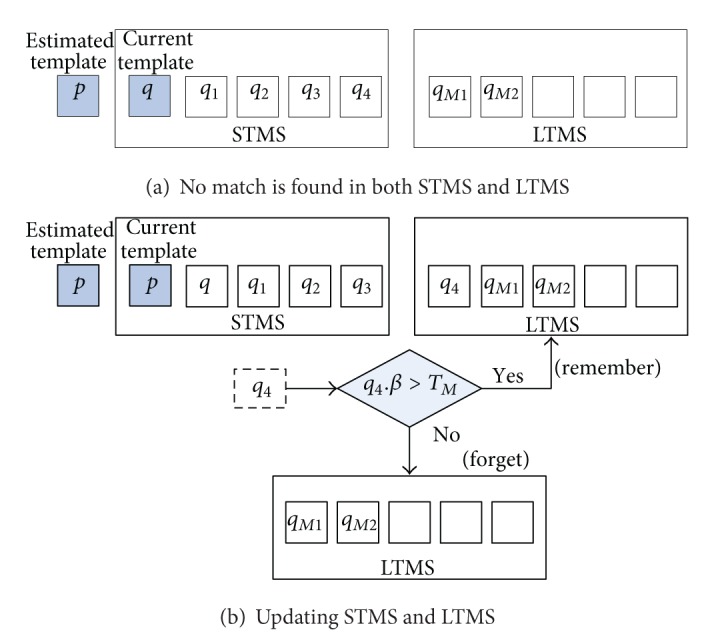
Illustration of updating STMS and LTMS when no match is found in both memory spaces.

If *ρ*[*p*, *q*_*Mj*_] ≤ *T*_*dl*_, it means that there is no any match in STMS and LTMS. The estimated template *p* is stored into STMS and used as the new object template (set *p*.*β* = 1), as seen in Figure 8. Meanwhile, if the STMS reaches its maximum capacity, remember or forget the oldest template in STMS (i.e., *q*_*K*_*s*_−1_) by the following substeps.

1. If *q*_*K*_*s*_−1_.*β* > *T*_*M*_ and the LTMS is full, forget the oldest template in LTMS (i.e., *q*_*M*__*K*_*l*__) and remember *q*_*K*_*s*_−1_.
2. If *q*_*K*_*s*_−1_.*β* ≤ *T*_*M*_, forget *q*_*K*_*s*_−1_.

As shown in Figure 8, when no match is found in both memory spaces, the current estimated template *p* is stored into STMS, while *q*_4_ (i.e., *K*_*s*_ − 1 = 4) is either remembered (*q*_4_.*β* > *T*_*M*_) or forgotten (*q*_4_.*β* ≤ *T*_*M*_).

Note that the templates in STMS and LTMS are stored in chronological order; that is, if a template is stored into STMS or LTMS earlier, it will move to the subsequent locations in order to make rooms for the newly reached templates.

## 4. Moving Object Tracking by MMAM

### 4.1. Object Detection and Modeling

To detect a color object, it is very important to obtain an effective color model to accurately represent and identify the object under various illumination conditions. In this paper, we use a histogram-based nonparametric modeling technique in YCbCr color space to model an object [32], which is much robust to lighting variations.

Giving the distribution of colors in an object region, let *px*_*i*,*j*_, be a pixel location inside the object region with the origin at the center of the object region, the non-parametric distribution of the object, *Q*, can be represented by the following [32]:

(14) $$\begin{array}{c} Q=\left\{q_{u};u=1,2,\ldots ,m\right\}, \end{array}$$

where

(15) $$\begin{array}{c} q_{u}=C\sum_{i=1,j=1}^{x,y}k\left({||px_{i,j}||}^{2}\right)\delta \left[b\left(px_{i,j}\right)-u\right], \end{array}$$

where *k* is the Epanechnikov kernel function, *δ* is the Kronecker delta function, and the function *b* : *R* ^2^ → {1,…, *m*} associates the pixel at location *px*_*i*,*j*_ with its color's index *b*(*px*_*i*,*j*_) in the histogram. The normalization constant *C* is derived by imposing the condition ∑_*u*=1_^*m*^ *q*_*u*_ = 1.

Suppose *P*_*y*_ is the non-parametric distribution of the candidate object at position *y* in the image, then the similarity or Bhattacharyya coefficient can be decided by the following [32]:

(16) $$\begin{array}{c} \rho \left(y\right)=\rho \left[P_{y},Q\right]=\sum_{u=1}^{m}\sqrt{p_{u}\left(y\right)q_{u}}. \end{array}$$

For tracking by agents, *ρ*(*y*) can be used to compute the fitness of an agent and the similarity coefficient between two appearance templates.

### 4.2. Implementation of the Tracking Algorithm

The memory-based multiagent model for object tracking can be described as follows.

**Step 1**

First locate the object in a video scene and then build the object appearance model by (14).

**Step 2**

**Figure 9:**
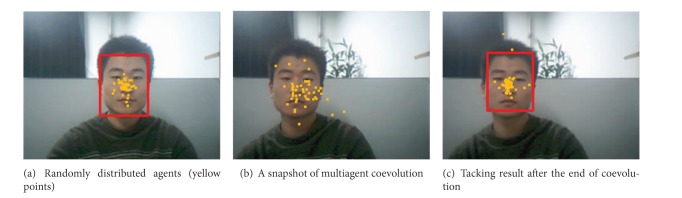
Object tracking by multiagent coevolution.

Randomly generate *N* × *N* agents near the located object region by adding a 2D Gaussian distribution *G*_*x*,*y*_(0,10), as shown in Figure 9(a), and then map the agents onto the 2D lattice-like environment.

**Step 3**

For each agent on the lattice, first retrieve the appearance template from its memory spaces, then compute the fitness of the agent, and then perform the competition, recombination, and migration behaviors when the object moves. A snapshot of multiagent coevolution is shown in Figure 9(b).

**Step 4**

Compute the final target by weighted averaging of all the agents on the lattice, and the tacking result after the end of coevolution is shown in Figure 9(c).

## 5. Experimental Results and Discussions

In this section, we aim to experimentally verify the efficacy of the proposed object tracking method. We compare the performance of the proposed method with the total model updating PF (TMU-PF) in practical tracking problems. We use some standard video sequences [33, 34] as testing dataset and the experiments are conducted on a computer with a P4 3.0 G Processor. 

It is worth noting that the parameters for the algorithms are set initially as follows in our experiments:

- *m* is the number of the bins for modeling the object using histogram and is set as *m* = 16 × 16;
- *T*_*dc*_ is used to measure the similarity between the estimated template and the current object template and is set as *T*_*dc*_ = 0.9;
- *T*_*ds*_ and *T*_*dl*_ are the thresholds used to find a match in STMS and LTMS, respectively, and are set as *T*_*ds*_ = *T*_*dl*_ = 0.8;
- *K*_*s*_ and *K*_*l*_ are the capacity of the STMS and LTMS, respectively, and are set as *K*_*l*_ = *K*_*s*_ = 5;
- *T*_*M*_ is a predefined threshold used to decide whether the template in STMS can be stored into LTMS or not and is initially set as *T*_*M*_ = 1;
- the total number of agents is 49; that is, the size of the lattice *A* is 7 × 7, the recombination probability *P*_*r*_ is 0.6, and the migration probability *P*_*m*_ is 0.05.
- the number of the particles used in particle filter-based tracking is set as 50 (almost equal to the number of agents used).

### 5.1. Tracking a Person with Large Appearance Change

**Figure 10:**
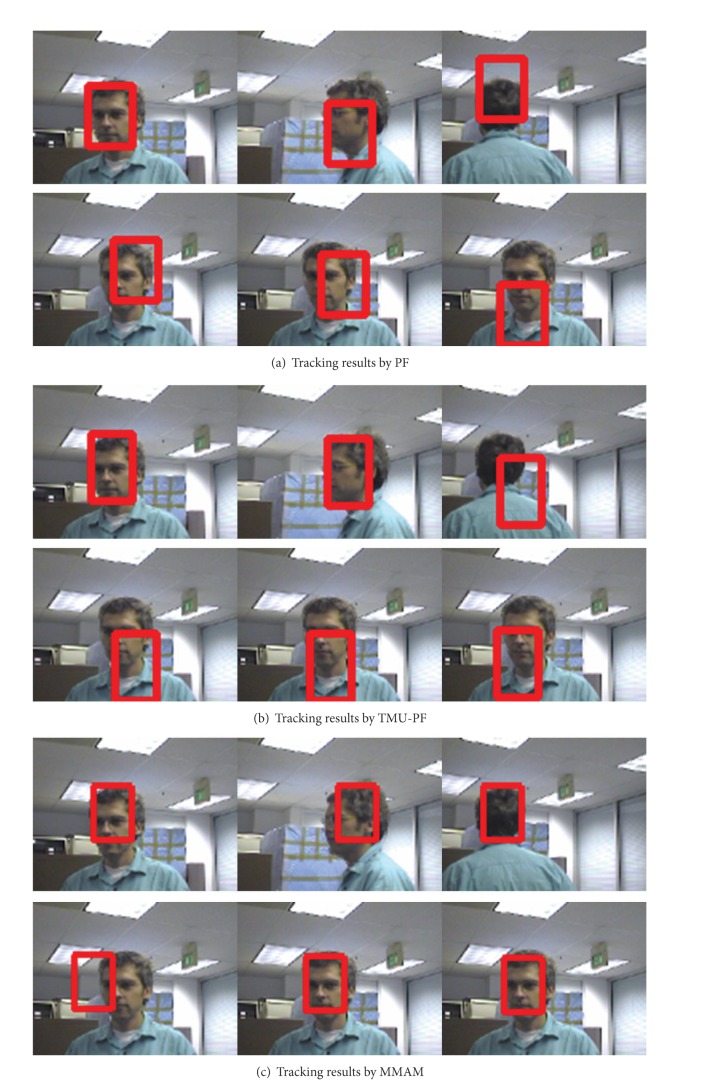
Tracking results of “seq_dk” sequence.

The first sets of experiments are to track a person with abrupt appearance changes. The video used in this experiment is clipped from the standard sequence “seq_dk” (The video sequences can be downloaded from http://www.ces.clemson.edu/~stb/research/headtracker/seq/) [33]. The tracking results of the man by traditional PF, TMU-PF, and the proposed method at frames 21, 58, 82, 83, 87, and 96 are shown in Figures 10(a), 10(b), and 10(c), respectively (the template is initialized manually). The human appearance changes very abruptly from frame 82 to frame 83. The results show that when the appearance is far from the initialized template, PF and TMU-PF deviate from the target gradually, while the original templates are remembered by the proposed method and when the appearance changes abruptly the relevant template can be recalled from the memory space of an agent.

**Figure 11:**
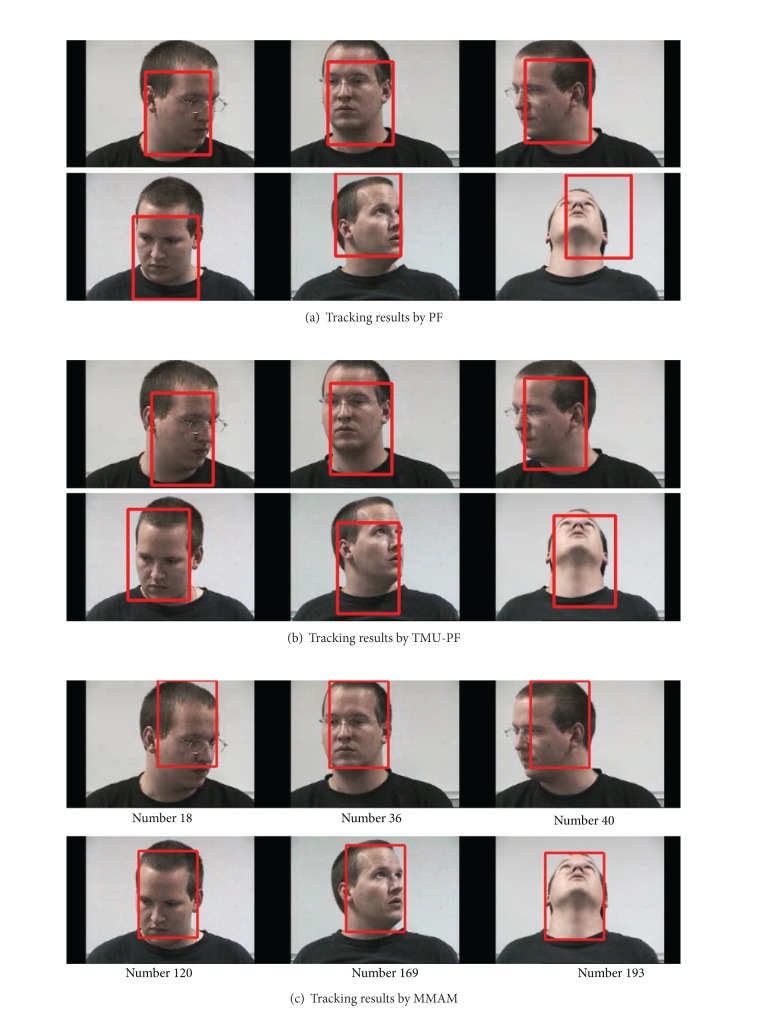
Tracking a person with pose changes.

Figure 11 displays experiments to track a person whose pose changes continuously in Head Pose Image Database (The video sequences can be downloaded from http://www-prima.inrialpes.fr/perso/Gourier/Faces/HPDatabase.html) [34]. Experimental results show that our proposed method can track more precisely than the other two methods.

### 5.2. Tracking a Person with Heavy Occlusions by Others

The second set of experiments aims at tracking persons who are occasionally occluded by another object.

**Figure 12:**
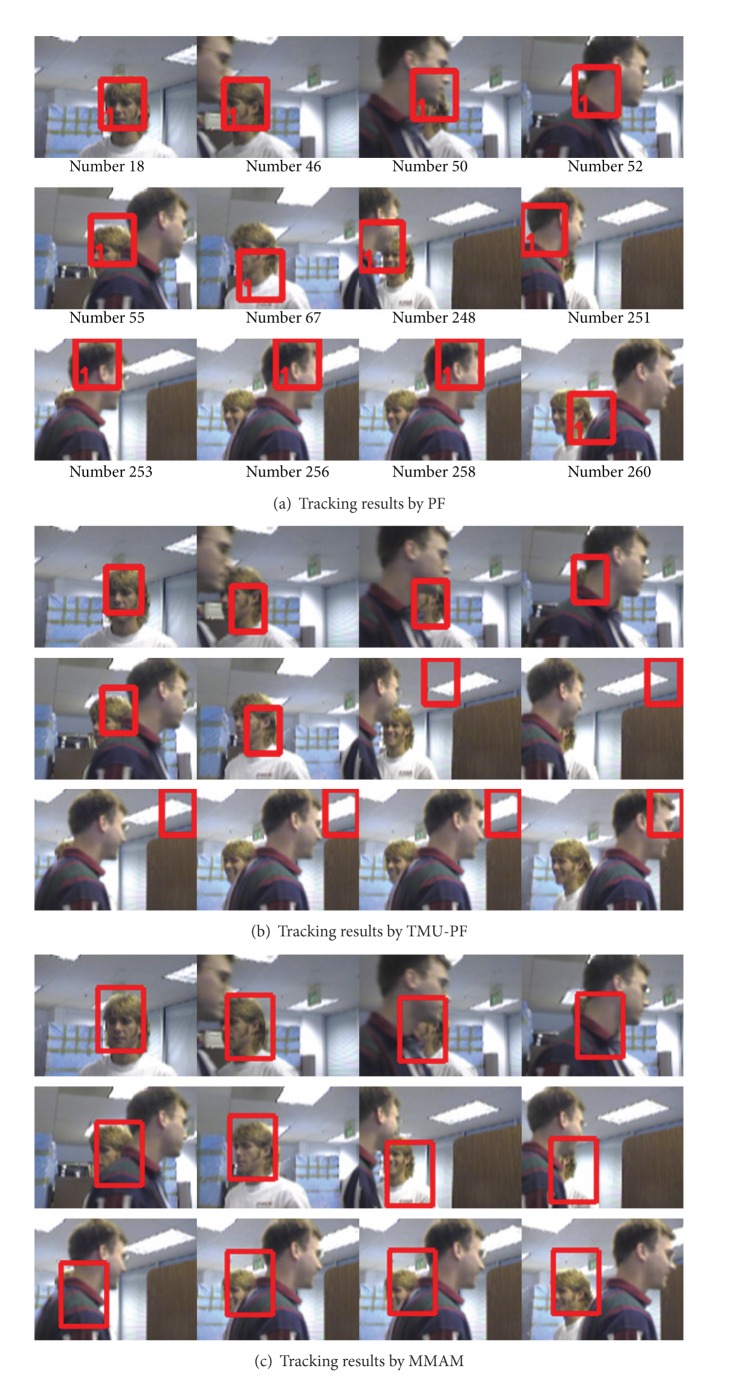
Tracking a fully occluded person.

The sequence used in the first experiment is also a standard sequence “seq_jd” [33]. In this sequence, the man is occluded twice by another person. The tracking results by PF, TMU-PF and the proposed MMAM are shown in Figures 12(a), 12(b), and 12(c), respectively (the template is initialized manually). It is worth noting that the man is totally occluded at frame 52 and frame 253. The results show that the proposed MMAM can still track the person correctly after recovered from the occlusion at frame 55 and frame 256.

**Figure 13:**
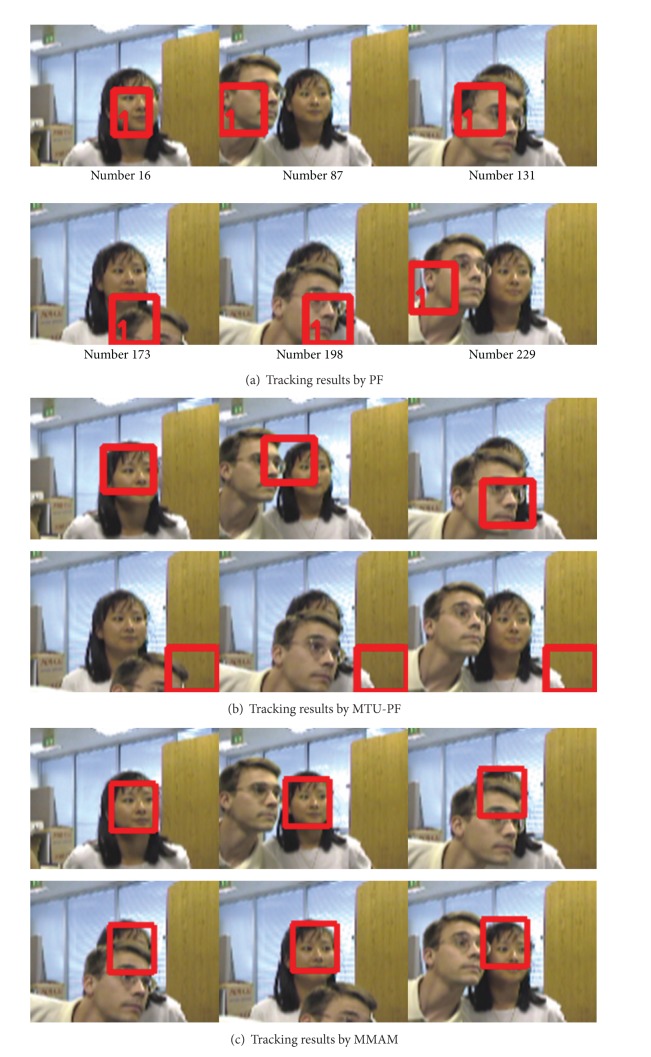
Tracking a fully occluded face.

Figure 13 shows the results of tracking a face which is fully occluded by another person (The templates are initialized manually).

Finally, unlike the particle filter-based tracking method, the proposed approach has no restrictions to the face moving direction and speed. The face will be located and tracked at any time.

## 6. Conclusions

In this paper, we propose a different approach for visual tracking inspired by the way human perceive the environment. A number of memory-based agents are distributed nearby the located object region and then mapped onto a 2D lattice-like environment for predicting the new location of the object by their coevolutionary behaviors, such as competition, recombination, and migration, which imitate the process when many people search for a target in real world. The three-stage of human brain memory model is incorporated into a multiagent coevolutionary process for finding a best match of the appearance of the object. Each agent can remember, retrieve, or forget the appearance of the object through its memory system by its own experience. Experimental results show that the proposed method can deal with large appearance changes and heavy occlusions when tracking a moving object. It can locate the correct object after the appearance changed or the occlusion recovered and outperforms the traditional particle filter based tracking.
